# Case Report: Aperiodic Fluctuations of Neural Activity in the Ictal MEG of a Child With Drug-Resistant Fronto-Temporal Epilepsy

**DOI:** 10.3389/fnhum.2021.646426

**Published:** 2021-03-04

**Authors:** Saskia van Heumen, Jeremy T. Moreau, Elisabeth Simard-Tremblay, Steffen Albrecht, Roy WR. Dudley, Sylvain Baillet

**Affiliations:** ^1^McConnell Brain Imaging Centre, Montreal Neurological Institute and Hospital, McGill University, Montreal, QC, Canada; ^2^Department of Pediatric Surgery, Division of Neurosurgery, Montreal Children’s Hospital, Montreal, QC, Canada; ^3^Cumming School of Medicine, University of Calgary, Calgary, AB, Canada; ^4^Department of Pediatrics, Division of Pediatric Neurology, Montreal Children’s Hospital, McGill University, Montreal, QC, Canada; ^5^Department of Pathology, Montreal Children’s Hospital, McGill University, Montreal, QC, Canada

**Keywords:** MEG (magnetoencephalography), epilepsy, pediatrics—children, neural dynamics, seizure, cortical networks

## Abstract

Successful surgical treatment of patients with focal drug-resistant epilepsy remains challenging, especially in cases for which it is difficult to define the area of cortex from which seizures originate, the seizure onset zone (SOZ). Various diagnostic methods are needed to select surgical candidates and determine the extent of resection. Interictal magnetoencephalography (MEG) with source imaging has proven to be useful for presurgical evaluation, but the use of ictal MEG data remains limited. The purpose of the present study was to determine whether pre-ictal variations of spectral properties of neural activity from ictal MEG recordings are predictive of SOZ location.We performed a 4 h overnight MEG recording in an 8-year-old child with drug-resistant focal epilepsy of suspected right fronto-temporal origin and captured one ~45-s seizure. The patient underwent a right temporal resection from the anterior temporal neocortex and amygdala to the mid-posterior temporal neocortex, sparing the hippocampus proper. She remains seizure-free 21 months postoperatively. The histopathological assessment confirmed frank focal cortical dysplasia (FCD) type IIa in the MEG-defined SOZ, which was based on source imaging of averaged ictal spikes at seizure onset. We investigated temporal changes (inter-ictal, pre-ictal, and ictal periods) together with spatial differences (SOZ vs. control regions) in spectral parameters of background brain activity, namely the aperiodic broadband offset and slope, and assessed how they confounded the interpretation of apparent variations of signal power in typical electrophysiological bands. Our data show that the SOZ was associated with a higher aperiodic offset and exponent during the seizure compared to control regions. Both parameters increased in all regions from 2 min before the seizure onwards. Regions anatomically closer to the SOZ also expressed higher values compared to contralateral regions, potentially indicating ictal spread. We also show that narrow-band power changes were caused by these fluctuations in the aperiodic component of ongoing brain activity. Our results indicate that the broadband aperiodic component of ongoing brain activity cannot be reduced to background noise of no physiological interest, and rather may be indicative of the neuropathophysiology of the SOZ. We believe these findings will inspire future studies of ictal MEG cases and confirm their significance.

## Introduction

Epilepsy is one of the most common and debilitating neurological disorders in children (Fiest et al., 2017), with one-third of patients unresponsive to pharmacotherapy (Geerts et al., 2010; Berg et al., 2014). In focal epilepsy, seizures arise from one region of the brain, whose surgical resection provides effective treatment (Wiebe et al., 2001; Engel et al., 2012). Epilepsy surgery is now recognized as the first-line, standard of care in well-selected pediatric patients (Dwivedi et al., 2017) with a clear seizure onset zone (SOZ) as the site of the primary organization of ictal discharges (Mountz et al., 2017). Well-defined cases with clearly visible lesions on magnetic resonance imaging (MRI), such as tumors, cavernous malformations, or focal cortical dysplasia (FCD) type IIb, have an 85–95% chance of seizure freedom after surgery (Colombo et al., 2012; Englot et al., 2016). However, poorly-defined cases (PDCs), which can be MRI-negative or have ill-defined signal abnormalities on MRI, such as FCD type IIa remain challenging and present considerably less positive surgical outcome (Colombo et al., 2012; Wang et al., 2016). For such PDCs, multimodal neuroimaging and in some cases, an invasive intracranial recording is required to identify the origin of seizures (Mountz et al., 2017). Nevertheless, and despite advances in neuroimaging and electrophysiology methods epilepsy surgical outcomes have not improved substantially. More research is needed to build a deeper understanding of how focal epilepsy affects large scale neural dynamics and assist with the clinical decision making of delineating the SOZ.

Magnetoencephalography (MEG) is now recognized as a valuable asset in the armamentarium of epileptologists and researchers. It allows for time-resolved source imaging of interictal spike waveforms and slow/fast oscillatory components of brain activity (Mountz et al., 2017; Choi and Wang, 2020; Stefan and Rampp, 2020), with reduced dependence on head tissue characteristics with respect to electroencephalography (EEG; Baillet, 2017). While an increasing number of studies have investigated the value of ictal MEG (Alkawadri et al., 2018), routine clinical MEG has so far mainly been used in the context of interictal measurements (Stefan and Rampp, 2020). This is in part due to the practical implications imposed by the multi-hour recordings often required to capture ictal events. However, we have recently shown that ictal MEG studies, and particularly overnight ictal MEG for patients with nocturnal epilepsy, can be reliably conducted and have the potential to improve SOZ identification (Moreau et al., 2020).

There are currently no widely accepted guidelines for the analysis and interpretation of ictal MEG data. Ictal spikes could in principle be analyzed akin to interictal events, however, with limited ability to map the Spatio-temporal evolution of epileptic activity after seizure onset. Spectral measurements of ongoing MEG source brain activity can be resolved in time and do not depend on an expert marking of discrete events. They, therefore, have the potential to reveal early, anatomically specific changes of neural dynamics during seizure initiation. Power spectrum density (PSD) estimates of electrophysiological brain signals typically reveal narrowband peaks that correspond to oscillatory signal components, superimposed on a broadband aperiodic profile traditionally discarded as background or brain “noise” (Haller et al., 2018). Studies of narrowband neurophysiological signals and their amplitude changes have been extensive in epilepsy research (Jacobs et al., 2012; Schönherr et al., 2017; Sueri et al., 2018).

There has been renewed interest in the objective characterization of the aperiodic spectral component and its implications in possible biased interpretations of narrowband signal changes (He, 2014). The power of the background spectral component decreases with frequency, as typical of scale-free brain dynamics, following a power-law function of the form: *Power* ~ 1/f^β^, with β a positive exponent (He, 2014). The aperiodic spectral component evolves with aging (Voytek et al., 2015) and neurodevelopment (He et al., 2019) and is affected in neurological disorders (Peterson et al., 2018) and by sedative drugs (Colombo et al., 2019). Yet, its functional significance concerning co-existing narrowband signals remains to be clarified. The 1/f^β^ component can readily be extracted from ongoing brain activity, without predefining frequency bands or specifying paroxysmal events (e.g., spikes). The profile of the aperiodic spectral component can be approximated from the parametric modeling of the PSD estimate derived from empirical signals. The two scalar parameters of the related parametric model are the spectral offset (y_offset_) and the slope, represented by the exponent (β; Haller et al., 2018). The offset accounts for the overall magnitude of the aperiodic spectral component, and the exponent accounts for its curved shape. A broadband increase of higher frequency power and/or decrease of lower-frequency power leads to a decreased slope. New practical analytical tools are available to fit these model parameters from the signal PSD. The resulting model fit can thereafter be removed from the PSD to investigate the actual narrowband oscillatory activity, if remaining, without possible confounds from the broadband arrhythmic activity (Haller et al., 2018).

Computational modeling and empirical data indicate that changes in the aperiodic slope are related to the synchrony of cell firing in neuronal networks, with an increased slope associated with increased population synchrony (Freeman and Zhai, 2009; Voytek and Knight, 2015; Voytek et al., 2015). The offset parameter has also been related to the aggregate of the underlying local neuronal spiking activity (Manning et al., 2009; Miller et al., 2009, 2013; Voytek and Knight, 2015). It has also been suggested that changes in excitation and inhibition properties of cell assemblies can produce shifts in the PSD slope (Gao et al., 2017; Peterson et al., 2018). One possible mechanism of epilepsy is hypothesized to be related to periods of excessive discharges combined with synchronous excitation of neuronal populations (Scharfman, 2007). Historically, hypersynchrony and hyperexcitability have been related to an imbalance in regional excitation/inhibition (E/I) *via* increased excitatory neurotransmitter conductance and/or disruption of mechanisms inhibiting firing (Khazipov, 2016; Shao et al., 2019). However, this view has been challenged as too limited, with additional possible mechanistic contributors to epileptogenesis being attributed to E/I imbalance, such as with synchronization *via* inhibition (Shao et al., 2019).

Here, we report on time-evolving changes of aperiodic broadband brain activity and oscillatory components of the power spectrum in an ictal MEG recording of a patient with focal drug-resistant epilepsy.

## Case Description

**Figure 1:**
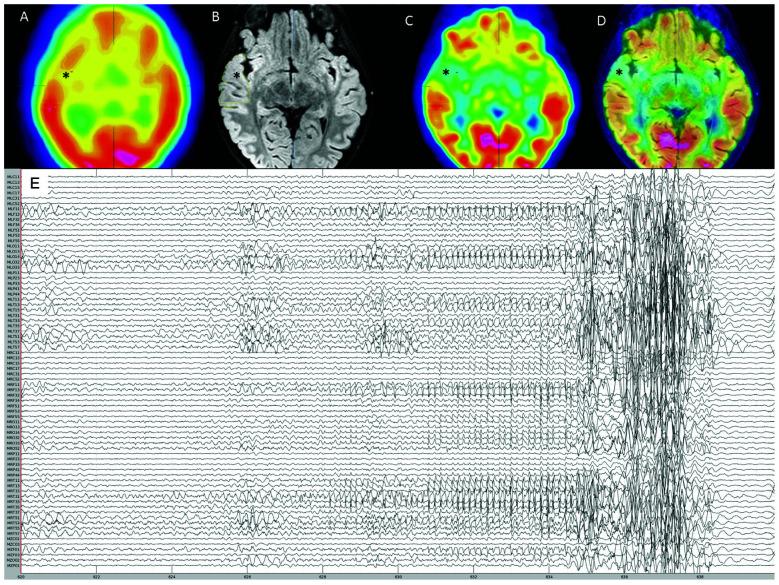
Pre-surgical evaluation neuroimaging. **(A)** Interictal SPECT showing right anterior temporal hypoperfusion (*). **(B)** 3T-magnetic resonance imaging (MRI) FLAIR sequence showing right anterior thickened cortex and blurring of gray matter-white matter junction (*). **(C)** FDG-PET showing right anterior temporal hypometabolism (*). **(D)** Co-registration of 3T-MRI FLAIR sequence with FDG-PET showing concordance between these imaging modalities (*). **(E)** Reduced MEG montage (76/275 MEG channels displayed) showing the seizure onset followed by movement artifact.

An 8-year-old girl with focal drug-resistant epilepsy was admitted for presurgical evaluation. Based on prior routine EEG and imaging studies (MRI, PET, SPECT), seizures were suspected to localize to the right fronto-temporal cortex (Figures 1A–D). At the time of admission, the patient was having about 10 seizures per day, which were characterized by brief (up to ~10 s) periods of unresponsiveness and a blank expression. Early MRIs showed only subtle asymmetry in temporal cortex sulci. Routine EEG showed frequent seizures over right fronto-temporal electrodes, mostly during sleep. Given the frequent nighttime seizures, we brought in the patient for an overnight MEG recording (~4 h of MEG with simultaneous EEG). During this recording, one ictal event was recorded (Figure 1E). Electrographic changes on MEG and EEG preceded clinical onset by about 6 s. Clinically, the seizure was characterized by arousal followed by a forced head version to the left. Ictal MEG source imaging localized to the right anterior temporal cortex and was concordant with source localization of interictal MEG spikes as well as subtle blurring at the gray-white matter junction on a 3T T1/FLAIR MRI. Interictal FDG-PET and SPECT were also concordant, localizing to the right temporal cortex. The patient underwent a tailored right temporal resection from the anterior portion of the temporal neocortex and amygdala to the mid-posterior temporal neocortex but sparing the hippocampus proper. She continues to be seizure-free at 22 months follow-up. Histopathological assessment of dedicated surgical sub-specimens confirmed frank FCD type IIa in the MEG-defined SOZ. Further clinical details and surgical pathology results are presented in Moreau et al. (2020).

## Methods

### MEG Data Acquisition and Analysis

MEG was acquired at the Montreal Neurological Institute (Montreal, Canada) using a 275-channel whole-head MEG system (CTF, Coquitlam, BC, Canada). In addition to MEG and EEG, Electrooculography (EOG) and electrocardiography (ECG) were simultaneously recorded during acquisition for eye blink and cardiac artifact detection. We captured a single ~45-s seizure during the 4-h overnight MEG sleep recording. MEG seizure identification was done according to the current clinical standards.

**Figure 2:**
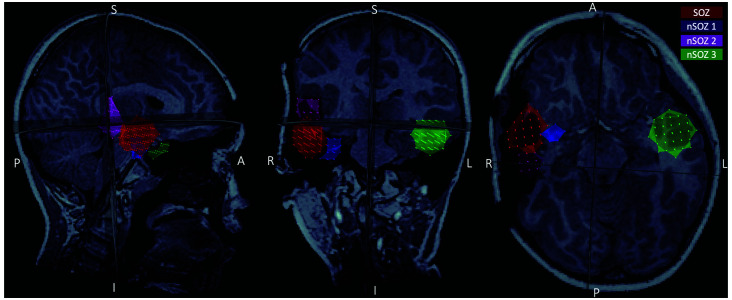
Postoperative MRI showing the four regions of interest (ROIs) used in the present study: the seizure onset zone (red), two ROIs in the resected region but outside the seizure onset zone (SOZ; pink and blue), and a homologous ROI contralateral to the SOZ (green).

We performed data analysis with Brainstorm^1^ (Tadel et al., 2011). Notch filtering at 60 Hz and its harmonics were used to remove artifacts from powerline frequency and a bandpass filter was applied to attenuate signals below 0.5 Hz and above 70 Hz. The data were resampled to 300 Hz. Cardiac artifacts were detected and removed using signal space projections (SSP). Interictal spike waves were included. Seizure onset was visually identified and consisted of a pattern of rhythmic alpha activity beginning over right temporal MEG sensors (Figure 1). Source imaging was computed using an unconstrained volumetric dSPM source model (Baillet et al., 2001) for better comparison with presurgical imaging and surgical margins on the postoperative MRI. We delineated the MEG SOZ based on source imaging of ten averaged peaks of rhythmic alpha oscillations at the ictal onset, which each localized to the same area. The results from surgical specimen histopathology confirmed the designated “MEG SOZ” due to presence of FCD in this region only. In addition, the fact that the patient went from having ~10 seizures/day to no seizures during 22 months follow-up, suggests that the true SOZ was resected in its entirety. Three additional non seizure onset zone (nSOZ) regions of interest (ROIs) were delineated. Namely, two ROIs outside the MEG SOZ but in the resected right temporal lobe (nSOZ_1_ and nSOZ_2_), which contained rare dysmorphic neurons (but not frank FCD) and one ROI in the contralateral hemisphere (nSOZ_3_), with homologous location and size to the SOZ’s ROI, but presumably devoid of epileptogenic pathology (i.e., no FCD or dysmorphic neurons). Figure 2 shows the ROIs on the postoperative MRI.

### Power Spectral Density and FOOOF Analysis

We derived the PSD using Welch’s method (Welch, 1967) as implemented in Brainstorm. The Fitting Oscillations and One-Over F (FOOOF) parametric model of the PSD (Haller et al., 2018) was obtained using the open-source FOOOF Python package (version 1.0.0) Brainstorm integration. We used the resulting PSDs to compute the offset and exponent of the aperiodic component within the range of 1–70 Hz for every ROI. Both parameters were estimated over 5-s consecutive epochs, from 10 min before to 15 s after seizure onset for all sources in the ROIs. We z-score normalized the resulting parameters with respect to the mean and standard deviation of a 15-min interictal recording collected >10 min before the ictal recording. To investigate relative differences between the four ROIs at every time point, we also calculated z-scores at every time point based on the mean and standard deviation across all sources in all ROIs. Lastly, we also calculated z-scores of the offsets and exponents for every epoch across the entire brain, for visualization purposes.

For PSD analyses, we extracted the mean power in the delta (1–4 Hz), theta (5–7 Hz), alpha (8–12 Hz), beta (15–29 Hz), and low-gamma (30–59 Hz) frequency bands for both the raw power spectrum and the power spectrum after removal of the aperiodic fit. Z-scores of the resulting values were computed at every time point from the mean and standard deviation of all sources in the four ROIs for the same time windows as for the aperiodic spectral component parameters.

## Results

Preprocessing of the data and removal of bad segments resulted in ~9 min of data before seizure onset, one 5-s epoch moments before seizure onset, one 5-s ictal epoch, and a post-ictal epoch shortly after seizure resolution. The interictal recording contained one interictal spike and the patient was in sleep stage N3 for most of the recording and sleep stage N2 from ~1 min before seizure onset. The data immediately following the ictal epoch was not analyzable due to movement artifact.

### Power Spectral Density Analysis: Aperiodic Component

**Figure 3:**
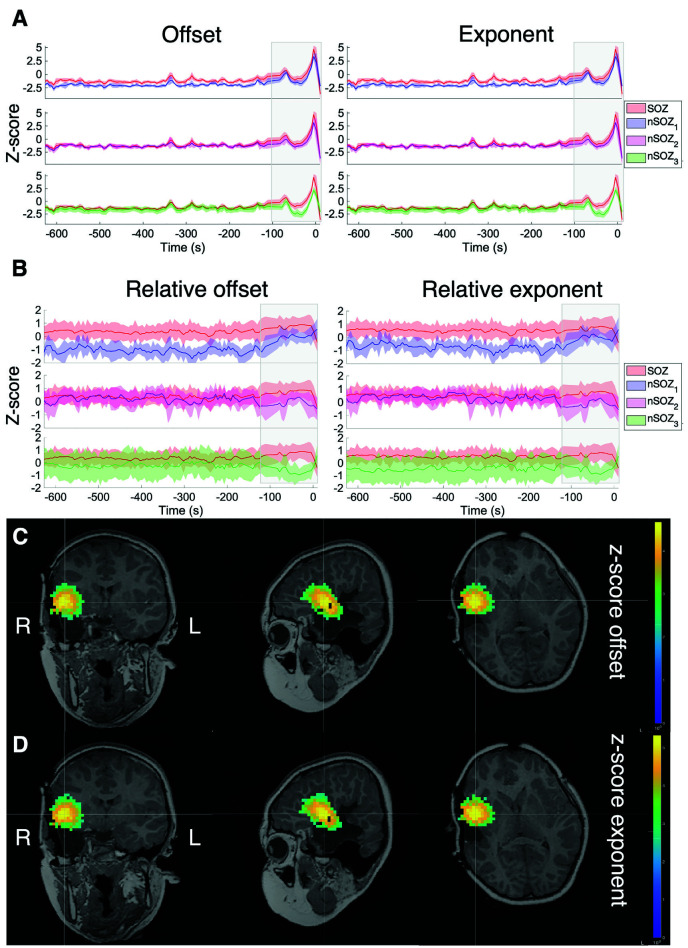
Z-score of the aperiodic offset and exponent before and during ictal onset, with seizure onset at *t* = 0. **(A)** Z-score normalized based on the mean and standard deviation of the prior interictal run (>10 min before seizure onset). **(B)** Z-score normalized based on the mean and standard deviation over all sources in all ROIs at every time point. **(C)** Z-score of the aperiodic offset for the entire brain over a 5-s mid-seizure epoch. **(D)** Z-score of the aperiodic exponent of the entire brain over a 5-s mid-seizure epoch.

Figure 3A shows the offset and exponent z-scores relative to the mean and standard deviation of the interictal recording. The offset and exponent fluctuated between 0.2 and 1.3 in the minutes before seizure onset (marked at *t* = 0). From ~100 s onwards, both values increased, reaching their peak during the seizure for all ROIs.

To clearly identify relative differences between the ROIs, z-scores were also computed across all ROIs at each time point (Figure 3B), which revealed visible differences between the SOZ, nSOZ_1_ and nSOZ_3_. Several minutes before seizure onset, both the offset and exponent were lower in the nSOZ_1_ compared to the SOZ. From ~100 s before seizure onset, these differences started to reduce. We observed the opposite phenomenon in the contralateral control ROI, with no clear differences with the SOZ long before the seizure and an increasing difference closer to seizure onset. We did not observe clear differences of both parameter values over the entire length of the recording between the SOZ and nSOZ_2_, both located in the resected brain region.

Figures 3C,D shows the results of the full-brain offset and exponent z-scores over the ictal epoch, plotted above the post-op MRI.

### Power Spectral Density Analysis: Narrow-Band Peaks

**Figure 4:**
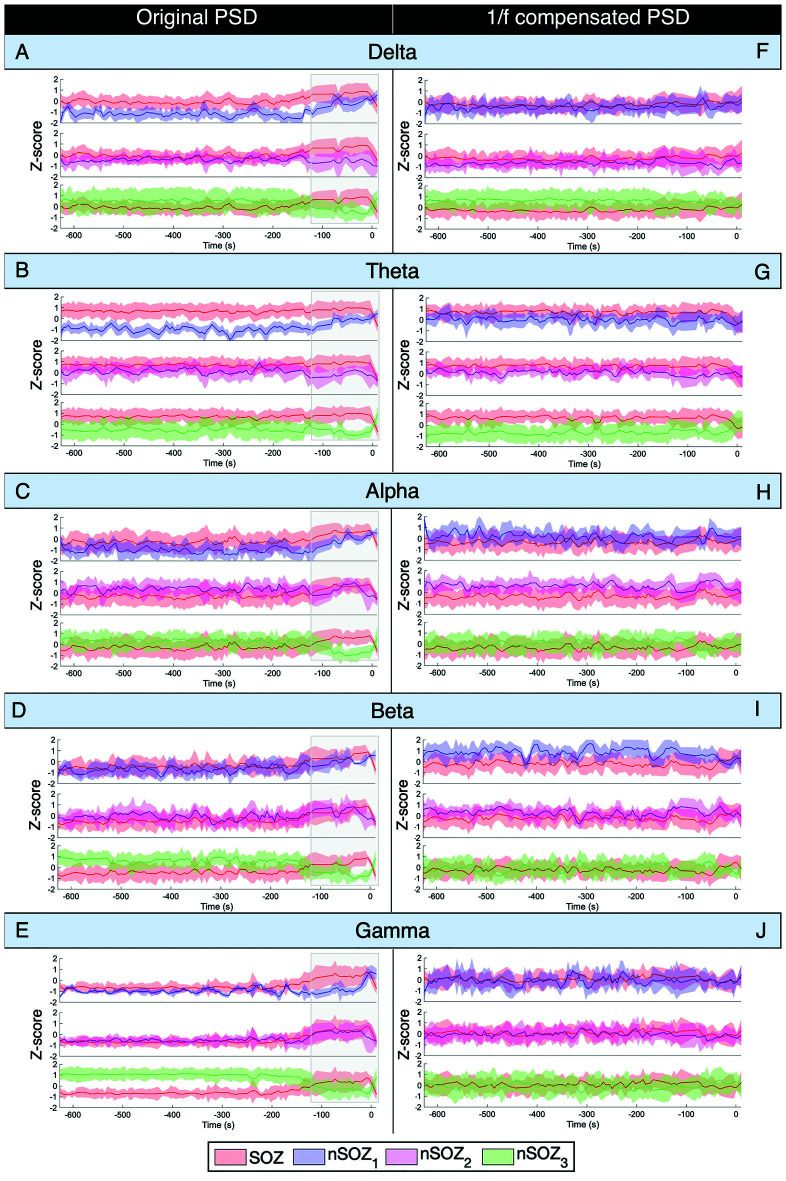
Power spectral density analysis of ictal MEG with seizure onset at * t* = 0. **(A–E)** Mean power Z-score for every frequency band for every 5-s epoch of the original non-compensated power spectrum. Z-score is based on the mean and standard deviation at every time point over all the ROIs. **(F–J)** Mean power Z-score for every frequency band for every 5-s epoch of the 1/f compensated power spectrum. Z-score is based on the mean and standard deviation at every time point over all the ROIs.

The results of the mean power of the raw PSD and 1/f-compensated PSD are shown in Figure 4. The non-compensated theta frequency band power of the original power spectrum showed remarkably similar patterns as the aperiodic parameters for all ROIs (Figure 4B). Indeed, higher power values were found in the SOZ until a couple of minutes before seizure onset, where differences between the SOZ and nSOZ_3_ (contralateral temporal lobe) increased, while they became smaller between the SOZ and nSOZ_1_. Further, there was no clear difference between SOZ and nSOZ_2_ over the entire recording. We observed similar patterns with the delta band power of the original PSD in right-lateralized ROIs (Figure 4A). However, delta power in the contralateral ROI was higher until ~100 s before seizure onset, at which point power dropped below SOZ values. Similar outcomes were obtained for power measures in the alpha (Figure 4C) and beta (Figure 4D) frequency bands of the original PSD, but the differences between SOZ and nSOZ_1_ were less evident. Lastly, power in the gamma frequency band of the original PSD showed no distinctive differences between the SOZ and nSOZ_2_ (Figure 4E). However, there were clear differences between SOZ, nSOZ_1_, and nSOZ_3_, especially starting ~110 s before seizure onset. Gamma power in the SOZ was higher than in nSOZ_1_ and nSOZ_3_ from ~110 s before seizure onwards, whereas SOZ power values were similar to those in nSOZ_1_ and much lower compared to nSOZ_3_ earlier in the recording.

After 1/f compensation of the power spectrum, these differences and temporal changes were no longer present in the alpha, beta, and gamma frequency bands (Figures 4H–J). On the other hand, delta power was lower and theta power was higher in the SOZ than in nSOZ_3_.

## Discussion

This case report presents an exploratory analysis of unique overnight ictal MEG recordings in a child with drug-resistant epilepsy. The ictal MEG SOZ was delineated and concordant with the surgical resection and confirmed FCD type IIa pathology. The patient remains seizure-free 22 months post-surgery. We investigated the dynamic changes in both the aperiodic broadband signals and narrow-band peaks of the power spectrum.

To our knowledge, no prior studies have examined temporal changes and spatial differences in the aperiodic power spectrum components of ictal MEG data. We found that both the offset and exponent parameters of the aperiodic fit increased shortly before the seizure and reached their highest values mid-seizure. This increase was observed in both the SOZ and control ROIs. However, values were relatively higher in the SOZ, especially as compared to the contralateral ROI (nSOZ_3_). We interpret these observations as possibly being related to rapid ictal spread to contiguous regions surrounding the SOZ (nSOZ_1_ and nSOZ_2_), which would not have impacted the contralateral hemisphere ROI (nSOZ_3_). However, it must be kept in mind that the influence of lower frequency dynamics, which is represented in the aperiodic component is more widespread and can therefore influence contiguous areas.

These results are compatible with the theory suggesting that increased aperiodic slope is related to hypersynchrony of oscillatory coupling, resulting in organized spiking (Manning et al., 2009; Miller et al., 2009, 2013; Voytek and Knight, 2015). This could manifest *via* increased synchrony in the SOZ immediately before and during seizures. Increased aperiodic signal power (offset), may reflect increased population spiking activity (Manning et al., 2009; Miller et al., 2009, 2013; Voytek and Knight, 2015). Note that the offset and slope are expected to be correlated measures (Haller et al., 2018). We do not expect that these changes are related to changes in sleep stages, since a decrease in slope would be expected from a transition between deeper sleep (N3) to lighter sleep (N1, N2; Lendner et al., 2019; Miskovic et al., 2019).

Spectral analysis in canonical frequency bands of both the original and 1/f compensated PSD showed that changes in power over time of the original PSD were similar to the patterns observed in aperiodic component parameters. Indeed, delta and theta power values were higher in the SOZ compared to nSOZ_1_ and this difference decreased from ~100 s before seizure onset. Furthermore, we did not observe clear differences between the SOZ and nSOZ_2_ at any time point or for any frequency band. Lastly, differences between the SOZ and the contralateral ROI also increased from ~120 s before ictal onset, with a higher power in the SOZ expressed in all frequency bands while power values were higher in the delta, beta, and gamma bands less than 120 s before seizure onset in the contralateral ROI. These findings suggest that such differences might be due to broadband changes, and not solely to changes in narrow-band oscillatory components.

Indeed, removal of the 1/f components from the PSD confirmed this hypothesis: most spatial differences (i.e., differences between ROIs) and temporal changes that consisted of narrow-band components disappeared after 1/f compensation. The only difference that remained was between the SOZ and the contralateral ROI in the delta and theta frequency bands. Oscillatory signal power was higher in the SOZ in the theta frequency band and vice versa for the delta band, although less pronounced.

These results show promise for SOZ delineation without the need for the definition of discrete events related to seizure onset and frequency bands of interest by an expert.

Some limitations include the limited amount of peri-ictal recording that could be analyzed due to extensive head movement artifacts that occurred shortly following electrographic seizure onset. Inter-ictal and pre-ictal data segments were substantially longer, providing more data samples and potentially more robust outcomes. This case was meant to illustrate novel applications of methods to more precisely characterize the spectral components of ictal MEG recordings. These results will need to be reproduced and further investigated in larger case series and with longer ictal recordings to better define the range of observable dynamic changes that can occur in the course of seizures.

## Conclusion

In this study, we provide early evidence suggesting that the broadband aperiodic component of the PSD contains useful neurophysiological information rather than just “background brain noise” and could contribute to helping define the SOZ. Studies with longer ictal MEG recordings will be needed to confirm and expand on the present findings. Overall, our data suggest that this novel approach holds promise for aiding in localizing the SOZ in children with focal epilepsy.

## Data Availability Statement

The raw data supporting the conclusions of this article will be made available by the authors, without undue reservation.

## Ethics Statement

The studies involving human participants were reviewed and approved by Research Ethics Board of the Montreal University Health Centre. Written informed consent to participate in this study was provided by the participants’ legal guardian/next of kin. Written informed consent was obtained from the minor(s)’ legal guardian/next of kin for the publication of any potentially identifiable images or data included in this article.

## Author Contributions

SH conducted the analysis and interpretation of the data, drafted and revised the manuscript for intellectual content. JM and RD contributed to the design and conceptualization of the study, a major role in the acquisition of data, analysis, interpretation of the data, and revised the manuscript for intellectual content. ES-T helped with marking the seizure and interpreting the EEG. SA analyzed the pathology specimens used for confirmation of the seizure onset zone. SB contributed to the design and conceptualization of the study, analysis, interpretation of the data and revised the manuscript for intellectual content. All authors contributed to the article and approved the submitted version.

## Conflict of Interest

The authors declare that the research was conducted in the absence of any commercial or financial relationships that could be construed as a potential conflict of interest. The reviewer GP declared a shared affiliation with the authors to the handling editor at time of review.

## Footnotes

^1^http://neuroimage.usc.edu/brainstorm

## References

[B1] Alkawadri R. Burgess R. C. Kakisaka Y. Mosher J. C. Alexopoulos A. V. (2018). Assessment of the utility of ictal magnetoencephalography in the localization of the epileptic seizure onset zone. JAMA Neurol. 75, 1264–1272. 10.1001/jamaneurol.2018.1430 29889930 PMC6233852

[B2] Baillet S. (2017). Magnetoencephalography for brain electrophysiology and imaging. Nat. Neurosci. 20, 327–339. 10.1038/nn.4504 28230841

[B3] Baillet S. Mosher J. Leahy R. (2001). Electromagnetic brain mapping. Signal. Process. Mag. IEEE 18, 14–30. 10.1109/79.962275

[B4] Berg A. T. Rychlik K. Levy S. R. Testa F. M. (2014). Complete remission of childhood-onset epilepsy: stability and prediction over two decades. Brain 137, 3213–3222. 10.1093/brain/awu294 25338950 PMC4240301

[B5] Choi J. Y. Wang Z. I. (2020). Merging magnetoencephalography into epilepsy presurgical work-up under the framework of multimodal integration. Neuroimaging. Clin. N. Am. 30, 249–259. 10.1016/j.nic.2020.01.005 32336411

[B6] Colombo M. Napolitani M. Boly M. Gosseries O. Casarotto S. Rosanova M.. (2019). The spectral exponent of the resting EEG indexes the presence of consciousness during unresponsiveness induced by propofol, xenon and ketamine. NeuroImage 189, 631–644. 10.1016/j.neuroimage.2019.01.024 30639334

[B7] Colombo N. Tassi L. Deleo F. Citterio A. Bramerio M. Mai R.. (2012). Focal cortical dysplasia type IIa and IIb: MRI aspects in 118 cases proven by histopathology. Neuroradiology 54, 1065–1077. 10.1007/s00234-012-1049-1 22695739

[B8] Dwivedi R. Ramanujam B. Chandra P. S. Sapra S. Gulati S. Kalaivani M.. (2017). Surgery for drug-resistant epilepsy in children. N. Engl. J. Med. 377, 1639–1647. 10.1056/NEJMoa1615335 29069568

[B9] Engel J. Jr. McDermott M. P. Wiebe S. Langfitt J. T. Stern J. M. Dewar S.. (2012). Early surgical therapy for drug-resistant temporal lobe epilepsy: a randomized trial. JAMA 307, 922–930. 10.1001/jama.2012.220 22396514 PMC4821633

[B10] Englot D. J. Chang E. F. Vecht C. J. (2016). Epilepsy and brain tumors. Handb. Clin. Neurol. 134, 267–285. 10.1016/B978-0-12-802997-8.00016-5 26948360 PMC4803433

[B11] Fiest K. M. Sauro K. M. Wiebe S. Patten S. B. Kwon C. - S. Dykeman J.. (2017). Prevalence and incidence of epilepsy: a systematic review and meta-analysis of international studies. Neurology 88, 296–303. 10.1212/WNL.0000000000003509 27986877 PMC5272794

[B12] Freeman W. J. Zhai J. (2009). Simulated power spectral density (PSD) of background electrocorticogram (ECoG). Cogn. Neurodyn. 3, 97–103. 10.1007/s11571-008-9064-y 19003455 PMC2645494

[B13] Gao R. Peterson E. J. Voytek B. (2017). Inferring synaptic excitation/inhibition balance from field potentials. NeuroImage 158, 70–78. 10.1016/j.neuroimage.2017.06.078 28676297

[B14] Geerts A. Arts W. F. Stroink H. Peeters E. Brouwer O. Peters B.. (2010). Course and outcome of childhood epilepsy: a 15-year follow-up of the dutch study of epilepsy in childhood. Epilepsia 51, 1189–1197. 10.1111/j.1528-1167.2010.02546.x 20557350

[B15] Haller M. Donoghue T. Peterson E. Varma P. Sebastian P. Gao R.. (2018). Parameterizing neural power spectra. BioRxiv [Preprint]. 10.1101/299859 PMC8106550 33230329

[B16] He B. J. (2014). Scale-free brain activity: past, present and future. Trends. Cogn. Sci. 18, 480–487. 10.1016/j.tics.2014.04.003 24788139 PMC4149861

[B17] He W. Donoghue T. Sowman P. F. Seymour R. A. Brock J. Crain S.. (2019). Co-increasing neuronal noise and beta power in the developing brain. BioRxiv [Preprint]. 10.1101/839258

[B18] Jacobs J. Staba R. Asano E. Otsubo H. Wu J. Y. Zijlmans M.. (2012). High-frequency oscillations (HFOs) in clinical epilepsy. Prog. Neurobiol. 98, 302–315. 10.1016/j.pneurobio.2012.03.001 22480752 PMC3674884

[B19] Khazipov R. (2016). GABAergic synchronization in epilepsy. Cold Spring Harb. Perspect Med. 6, a022764–a022764. 10.1101/cshperspect.a022764 26747834 PMC4743071

[B20] Lendner J. D. Helfrich R. F. Mander B. A. Romundstad L. Lin J. J. Walker M. P.. (2019). An electrophysiological marker of arousal level in humans. BioRxiv [Preprint]. 10.1101/625210 PMC7394547 32720644

[B21] Manning J. Jacobs J. Fried I. Kahana M. (2009). Broadband shifts in local field potential power spectra are correlated with single-neuron spiking in humans. J. Neurosci. 29, 13613–13620. 10.1523/JNEUROSCI.2041-09.2009 19864573 PMC3001247

[B22] Miller K. J. Sorensen L. B. Ojemann J. G. den Nijs M. (2009). Power-law scaling in the brain surface electric potential. PLoS Comput. Biol. 5, e1000609–e1000609. 10.1371/journal.pcbi.1000609 20019800 PMC2787015

[B23] Miller K. Honey C. Hermes D. Rao R. Dennijs M. Ojemann J. (2013). Broadband changes in the cortical surface potential track activation of functionally diverse neuronal populations. NeuroImage 85, 711–720. 10.1016/j.neuroimage.2013.08.070 24018305 PMC4347924

[B24] Miskovic V. MacDonald K. J. Rhodes L. J. Cote K. A. (2019). Changes in EEG multiscale entropy and power-law frequency scaling during the human sleep cycle. Hum. Brain Mapp. 40, 538–551. 10.1002/hbm.24393 30259594 PMC6865770

[B25] Moreau J. T. Simard-Tremblay E. Albrecht S. Rosenblatt B. Baillet S. Dudley R. W. R.. (2020). Overnight ictal magnetoencephalography. Neurol. Clin. Pract. 10.1212/CPJ.0000000000000937 PMC8610549 34840892

[B26] Mountz J. M. Patterson C. M. Tamber M. S. (2017). Pediatric epilepsy: neurology, functional imaging and neurosurgery. Semin. Nucl. Med. 47, 170–187. 10.1053/j.semnuclmed.2016.10.003 28237005

[B27] Peterson E. J. Rosen B. Q. Campbell A. M. Belger A. Voytek B. (2018). 1/f neural noise is a better predictor of schizophrenia than neural oscillations. BioRxiv [Preprint]. 10.1101/113449 PMC11905180 37287239

[B29] Scharfman H. E. (2007). The neurobiology of epilepsy. Curr. Neurol. Neurosci. Rep. 7, 348–354. 10.1007/s11910-007-0053-z 17618543 PMC2492886

[B30] Schönherr M. Stefan H. Hamer H. M. Rössler K. Buchfelder M. Rampp S.. (2017). The delta between postoperative seizure freedom and persistence: automatically detected focal slow waves after epilepsy surgery. NeuroImage Clin. 13, 256–263. 10.1016/j.jsp.2020.12.001 28018852 PMC5167245

[B31] Shao L. - R. Habela C. Stafstrom C. (2019). Pediatric epilepsy mechanisms: expanding the paradigm of excitation/inhibition imbalance. Children 6:23. 10.3390/children6020023 30764523 PMC6406372

[B32] Stefan H. Rampp S. (2020). Interictal and Ictal MEG in presurgical evaluation for epilepsy surgery. Acta Epileptol. 2:11. 10.1186/s42494-020-00020-2

[B33] Sueri C. Gasparini S. Balestrini S. Labate A. Gambardella A. Russo E.. (2018). Diagnostic biomarkers of epilepsy. Curr. Pharm. Biotechnol. 19, 440–450. 10.2174/1389201019666180713095251 30003857

[B34] Tadel F. Baillet S. Mosher J. C. Pantazis D. Leahy R. M. (2011). Brainstorm: a user-friendly application for MEG/EEG analysis. Comput. Intell. Neurosci. 2011:879716. 10.1155/2011/879716 21584256 PMC3090754

[B35] Voytek B. Knight R. T. (2015). Dynamic network communication as a unifying neural basis for cognition, development, aging and disease. Biol. Psychiatry 77, 1089–1097. 10.1016/j.biopsych.2015.04.016 26005114 PMC4443259

[B36] Voytek B. Kramer M. A. Case J. Lepage K. Q. Tempesta Z. R. Knight R. T.. (2015). Age-related changes in 1/f neural electrophysiological noise. J. Neurosci. 35, LP13257–LP13265. 10.1523/JNEUROSCI.2332-14.2015 26400953 PMC4579381

[B37] Wang X. Zhang C. Wang Y. Hu W. Shao X. Zhang J.. (2016). Prognostic factors for seizure outcome in patients with MRI-negative temporal lobe epilepsy: a meta-analysis and systematic review. Seizure 38, 54–62. 10.1016/j.seizure.2016.04.002 27182689

[B38] Welch P. (1967). The use of fast fourier transform for the estimation of power spectra: a method based on time averaging over short, modified periodograms. IEEE Trans. Audio Electroacoust. 15, 70–73. 10.1109/TAU.1967.1161901

[B39] Wiebe S. Blume W. Girvin J. Eliasziw M. A. (2001). Randomized, controlled trial of surgery for temporal-lobe epilepsy. N. Engl. J. Med. 345, 311–318. 10.1056/NEJM200108023450501 11484687

